# One-Year Results of Photorefractive Keratectomy for Myopia and Compound Myopic Astigmatism with 210 nm Wavelength All Solid-State Laser for Refractive Surgery

**DOI:** 10.3390/jcm12134311

**Published:** 2023-06-27

**Authors:** Anna M. Roszkowska, Giuseppe Tumminello, Carmelo Licitra, Alice A. Severo, Leandro Inferrera, Umberto Camellin, Domenico Schiano-Lomoriello, Pasquale Aragona

**Affiliations:** 1 Ophthalmology Clinic, Department of Biomedical Sciences, University of Messina, 98100 Messina, Italy; 2 Department of Ophthalmology, Andrzej Frycz Modrzewski Krakow University, 30-705 Krakow, Poland; 3 Eye Clinic, Department of Medical, Surgical Sciences and Health, University of Trieste, 34127 Trieste, Italy; 4 IRCCS–Fondazione Bietti, 00198 Rome, Italy

**Keywords:** solid-state laser, 210 nm wavelength, refractive surgery, photorefractive keratectomy

## Abstract

Background: To examine the 12-month clinical and refractive outcomes of PRK performed with a UV all-solid-state laser. Methods: The study included healthy patients with myopia and/or compound myopic astigmatism enrolled for refractive surgery and treated with PRK using a 210 nm wavelength, 2 kHz repetition rate, UV all-solid-state laser (LaserSoft, Katana Technologies GmbH, Kleinmachnow, Germany). All subjects were examined at baseline and after 1, 3, 6, and 12 months after the treatment with a slit lamp, refraction, visual acuity assessment (logMAR chart), tonometry, ophthalmoscopy, and corneal tomography with a Scheimpflug camera. The outcome measures considered were uncorrected (UDVA) and corrected (CDVA) distance visual acuities, refraction, central corneal thickness, and transparency. The efficacy, safety, predictability, and stability were determined. Results: The study included 34 eyes of 19 patients. The mean UDVA changed from 1.20 ± 0.43 to −0.05 ± 0.10 logMAR at 12 months, and the mean CDVA changed from −0.03 ± 0.06 to −0.06 ± 0.09 logMAR, respectively. The mean spherical equivalent (SE) changed from −4.90 ± 2.12 D to −0.01 ± 0.40 D and was within ±0.50 D of the intended correction in 91% of eyes and within ±1.00 D in 97% of eyes at 12 months. No eyes lost lines of visual acuity, and 64% of eyes gained one or more lines. Conclusions: PRK with the 210 nm wavelength, 2 kHz repetition rate, all-solid-state laser LaserSoft system proved to have good visual, refractive, and clinical outcomes after the follow-up at 12 months. The emerging gas-free, solid-state technology might be considered a valid alternative for the gas operating lasers for corneal refractive surgery.

## 1. Introduction

Laser vision correction with excimer lasers as ultraviolet sources has been successfully performed for over 30 years, and technological improvement has permitted the achievement of excellent efficacy, safety, and predictability. In the meantime, solid-state laser technology has become a more reliable source for treating organic tissue materials, and the possibility of performing corneal ablation with high efficacy and safety as compared to using an excimer laser is challenging, and has been investigated in experimental studies with good results [1,2,3,4,5]. Since the beginning, corneal refractive surgery with a solid-state laser has been a safe and effective procedure [2,6,7,8,9,10,11,12,13,14,15,16,17,18,19,20,21,22,23]. Now, growing interest has been noted with the recent presentation of the new platform [24].

The advantage of the LaserSoft solid-state laser platform and solid-state technology is related to the high pulse-to-pulse stability, small spot size, and high repetition rate. Contrary to excimer lasers, the noise level during operation is significantly less, and the system operates in nearly total silence, making the procedure comfortable for patients. Additionally, the hydration of the tissue needs to be controlled for the excimer lasers working at 193 nm, as it might affect the ablation process and, consequently, clinical results, whereas solid-state lasers emitting longer wavelengths are not dependent on corneal hydration and operating theater humidity [18,25,26]. Furthermore, due to the absence of gas, the costs of solid-state laser maintenance are lower when compared to excimer lasers.

The purpose of the present study was to investigate the clinical and refractive outcome of PRK performed with a 210 nm wavelength, 2 kHz repetition rate, all-solid-state laser system for refractive surgery (LaserSoft, Katana Technologies GmbH, Kleinmachnow, Germany) in patients with myopia and myopic compound astigmatism, with follow-up after 12 months.

## 2. Materials and Methods

In this retrospective observational study, 34 eyes of 19 patients (12M and 7F) treated with PRK with solid-state laser platform (LaserSoft) were examined.

The initial sample consisted of 28 patients with a total of 51 eyes. Nine patients were excluded from the study because they did not complete the follow-up. A total of 4 subjects decided to be followed up by their ophthalmologists in other centers; 3 completed only 1 month of control, and 2 patients completed only 3 months of control. They were ruled out from the study due to the COVID-19 pandemic and difficulties in moving from their place of residence.

Only patients who completed all follow-up controls at 1, 3, 6, and 12 months were considered. The exclusion criteria for laser refractive surgery comprised unstable refraction (changes in last two years prior to surgery), evidence of ocular surface or/and corneal disease (e.g., dry eye, keratoconus, corneal dystrophies, or degenerations), previous ocular surgery, history of glaucoma or ocular trauma, congenital or acquired corneal and lens opacities, and any systemic disease likely to alter corneal healing. Patients with a history of corneal and systemic autoimmune pathologies were excluded.

The clinical examination comprised the uncorrected (UDVA) and corrected (CDVA) visual acuity assessment using the logMAR chart, refraction, slit lamp evaluation, tonometry, and ophthalmoscopy. The corneal parameters were analyzed with corneal topography (Antares, CSO, Scandicci (FI), Italy) and Scheimpflug tomography (Pentacam, Oculus Systems, Wetzlar, Germany). All patients were examined before and after 1, 3, 6, and 12 months after the refractive procedure. Contact lens use was suspended 2 weeks before the preoperative measurements.

The main outcome measures considered for evaluation were UDVA, CDVA, refraction, and corneal transparency. The presence of haze was recorded according to Fantes’ classification [27].

Table 1 shows the demographic characteristics of studied patients.

The study followed the tenets of the Declaration of Helsinki and was approved by the Ethical Committee of the University Hospital of Messina.

### 2.1. Surgical Technique

Written informed consent was obtained from all patients before the laser procedure.

Anesthetic drops (Oxybuprocaine hydrochloride 0.4%, Laboratoires Thea, Clermont Ferrand, France) were instilled and 20% ethyl alcohol was applied for 20 s, and corneal epithelium with diameter of 9 mm was removed with a blunt spatula. The PRK was performed using Standard Visual Acuity Correction (VAC) aspheric ablation pattern by all-solid-state Q-switched, frequency-shifted laser (LaserSoft, Katana Technologies, Kleinmachow, Germany) working with a Gaussian spot with diameter of 0.2 mm and repetition rate of 2 kHz, and using an eye tracker with latency of 1 millisecond.

All PRK procedures were performed by one experienced surgeon (A.M.R.), with the ablation zone varying between 6.5 and 7.0 mm with a 1 mm of transition. The optical zone was planned according to the size of the scotopic pupil of each patient. The laser correction was centered on visual axis. Mitomycin C was not used after ablation, and a soft contact lens was applied after the treatment.

Therapy with drops of steroid and antibiotic agents (dexamethasone 0.1% + netilmicin 0.3%) and preservative-free artificial tears (0.3% trehalose + 0.15% hyaluronate sodium) to be applied 4 times daily for 5 days was prescribed until the epithelium healed. Then, the contact lenses were removed, and corticosteroid drops (loteprednol etabonate 0.5%) to be applied 4 times daily for 10 days and then 3 times successively daily for 1 month were prescribed, while the artificial tears were continued 4 times daily for 3 months.

### 2.2. Statistical Analysis

Descriptive statistics were provided to describe the characteristics of the studied population. Analysis was performed using Microsoft Excel software (2019, Microsoft Corp. Redmond, USA), and the data were plotted in sets of six standard graphs that summarized efficacy, predictability, safety, magnitude of refractive astigmatism, and stability accordingly to the standardized graphs and terms for refractive surgery results [15].

Efficacy was evaluated using the UCVA values at 12 months. Efficacy index was calculated as ratio between postoperative UDVA and preoperative CDVA.

Safety assessment was performed by analysis of gained and lost lines of CDVA, and the safety index was calculated as ratio between postoperative CDVA and preoperative CDVA.

Accuracy was evaluated by linear regression line obtained with plotting attempted versus achieved SE.

Stability of the procedure was assessed by analysis of mean SE after 1, 3, 6, and 12 months after the treatment.

## 3. Results

The 34 eyes of 19 patients (12M and 7F) with a mean age of 34.32 ± 8.27 (range 21 to 52 years) were examined.

**Figure 1:**
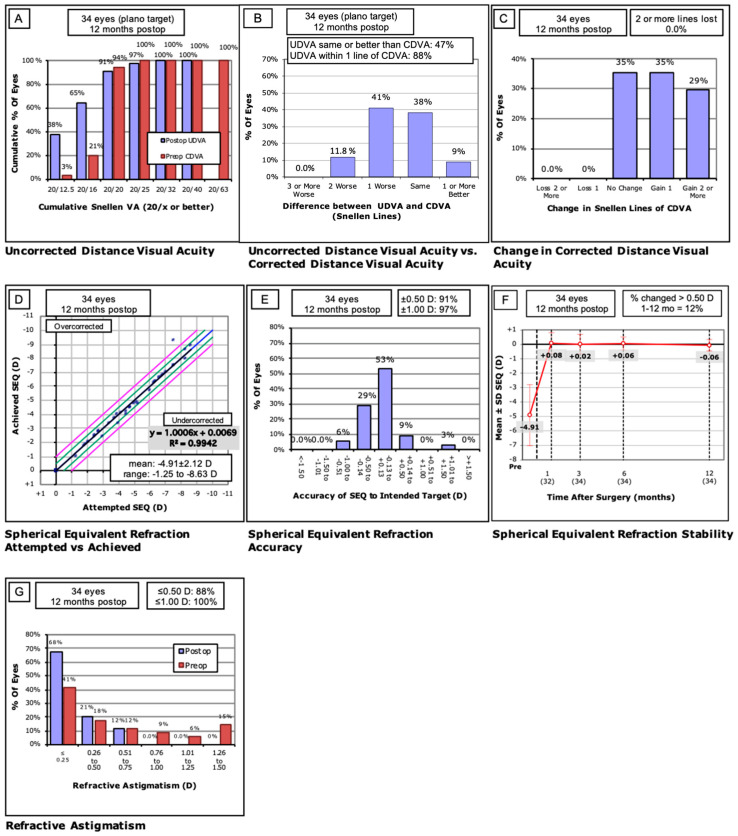
Visual outcomes after photorefractive keratectomy (PRK) with solid-state laser (LaserSoft, Katana Technologies GmbH, Kleinmachnow, Germany). (**A**) Cumulative 12-month postoperative uncorrected distance visual acuity (UDVA) and preoperative corrected distance visual acuity (CDVA). (**B**) Changes in Snellen lines of preoperative and postoperative CDVA. (**C**) Attempted vs. achieved spherical equivalent refraction. (**D**) The accuracy of spherical equivalent refraction (SEQ) to the intended target at 12 months after surgery. Black line: equality. Space between the two green lines: error range within 0.5D. Space between the two purple lines: error range within 1D. (**E**) Comparative distribution of preoperative and 12-month postoperative refractive cylinder. (**F**) Stability of spherical equivalent refraction at 1, 3, 6, and 12 months after surgery. (**G**) Percentages of preoperative and postoperative refractive cylinder at 12 months.

The results are represented in standard graphs for reporting outcomes in refractive surgery (Figure 1) [15].

### 3.1. Efficacy

The mean preoperative UDVA was 1.20 ± 0.43 (range 1.70 to 0.05) logMAR, and it changed to −0.05 ± 0.10 (range 0.2 to –0.2) logMAR after 12 months. The mean CDVA changed from −0.03 ± 0.06 (range 0.10 to −0.20) logMAR to −0.06 ± 0.09 (range 0.18 to −0.2) after the treatment.

The efficacy index (postoperative UDVA/preoperative CDVA) was 1.16 (Figure 1A).

### 3.2. Safety

The safety index (postoperative CDVA/preoperative CDVA) was 1.15.

No patient lost one or more lines of CDVA. Figure 1C shows the changes in CDVA lines (Figure 1C). No vision-threatening complications occurred during surgery or the postoperative period. No patient reported halos or glare disturbances. No retreatment was performed. A haze of grade 1 was registered in 11.8% of eyes at 1–3 months and was not observed after the follow-up at 12 months.

### 3.3. Accuracy and Predictability

Twelve months after surgery, 91% of eyes were within ±0.50 D of attempted correction, and 97% were within ±1.00 D. Figure 1D shows the scatterplot of the attempted versus achieved correction. Figure 1F shows the difference in SE between the intended and achieved correction as a function of time.

As to the magnitude of astigmatism, at the end of the evaluation period, 88% of eyes were within 0.50 D, and 100% were within ±1 D of cylinder (Figure 1G).

### 3.4. Refraction

The mean sphere changed from −4.90 ± 2.11 (range −8.50 to −1.00) D to 0.00 ± 0.35 (range −0.75 to 1.00) D after 12 months; the mean preoperative magnitude of refractive astigmatism was −0.68 ± 0.87 (range −4.00 to 0.00) D, and it changed to −0.03 ± 0.55 (range −1.50 to 1.50) D after 12 months. Spherical equivalent refraction changed from −4.90 ± 2.12 (range −8.63 to −1.25) D to −0.01 ± 0.40 (−0.75 to 1.25) D after 12 months.

### 3.5. Stability

Good stability was registered during the 12-month follow-up period, as shown in Figure 1F. The mean SE was +0.08 D after 1 month, and it was −0.06 D at 12 months.

## 4. Discussion

Laser refractive surgery is considered a safe and effective procedure that has been performed worldwide for about three decades [14]. Particularly, excimer lasers have reached a high level of technological improvement resulting in refined corneal shaping algorithms with wide optical zones and transition working with reduced spot diameter and increased frequency [14,17].

However, excimer lasers use an argon fluoride gas as the ultraviolet source, which is considered toxic and must be constantly exchanged, raising the costs of maintenance.

Solid-state lasers are characterized by different technical properties that make them remarkably interesting to be considered as an alternative platform for refractive surgery procedures. The advantages of solid-state technology comprise lower costs of maintenance as compared to excimers, no gas ablation, the procedure not being influenced by liquids on the corneal surface, and treatment being possible without drying the cornea, as the solid-state radiation is not absorbed by water.

The solid-state laser used in our study presents technical features that differentiate it from excimer lasers. It is characterized by a small laser spot of 0.2 mm with a Gaussian profile, a high repetition rate of 2 kHz, and a silent working mode that is very comfortable for the patients. An important aspect of the small spot diameter is the considerably lower mechanical stress caused by the acoustic shock wave generated in the ablation process. Krueger et al. measured these acoustic shock waves and stated that their amplitude increases with increasing spot diameter [28]. Kermani and Lubatschowski reported that the mechanical stress involved in laser-induced acoustic shock waves may produce cellular alterations that damage the collagen structure [29]. In the solid-state laser, the high repetition rate and the low energy per pulse result in a completely silent procedure and no audible acoustic waves, such as those generated during excimer laser treatments. In our experience, the low noise produced by the laser makes patients more comfortable, with increased compliance during the procedure. Future studies regarding the intraoperative and postoperative comfort of patients treated with solid-state laser PRK are needed to confirm our clinical observations.

As previously stated, the solid-state radiation at 210 nm is not absorbed by water and, thus, is not influenced by liquids on the corneal surface [16]. Thus, the fluids on the corneal surface and environmental humidity variations have no effects on the laser beam and ablation process; the treatment probably has less thermal stress and consequently less inflammation, and has already reported clinically registered faster corneal healing [2,9,18]. Furthermore, the platform does not require the use of nomograms. The gas-free lasers require less maintenance, with a relative reduction in costs due to the argon gas management, and the diode pump system is characterized by long-lasting effectiveness. The literature related to solid-state lasers comprises both experimental and clinical studies [1,2,3,4,5,6,7,8,9,10,11,12,13,18,19,20,21,22,23,24]. In the studies on murine corneas, Ren et al. and Tsiklis et al. demonstrated similar histopathologic responses to solid-state laser and excimer laser ablations in murine corneas [1,4]. Sanders et al. compared the effects of excimer and solid-state laser radiations on the murine cornea and demonstrated a higher level of superoxide dismutase in the 213 nm laser, suggesting a better endogenous response of the stromal cells to the laser-induced free radical production. Additionally, the authors observed that the 213 nm radiation resulted in a more safe and predictable approach as compared to the effects of the 193 nm laser [7].

The majority of clinical studies report results of refractive surgery performed with two solid-state lasers [2,8,9,10,11,12,13,18,19,20,21,22,23]. The LaserSoft (Katana Technologies, Kleinmachow, Germany), which operates at a 210 nm wavelength, 0.2 mm diameter Gaussian spot, and repetition rate of 2 kHz, and the Pulzar Z1 (Customvis, Perth, Australia), working with a 213 nm laser and a 0.6 mm spot with a repetition rate of 300 Hz. Single reports regard two other platforms such as the Novatec Light Blade, no longer in use, and the Russian OLIMP-2000/213–300 Hz system [6,23]. Recently a new platform, Aquarius Z solid-state (Ziemer Ophthalmic Systems AG, Port, Switzerland), was presented with preliminary clinical data on three patients, confirming the clinical and technological benefits and the growing interest in solid-state technology [24].

The reports on PRK performed with a laser at 210 nm showed good clinical results for myopia and compound myopic astigmatism [2,6]. In our previous paper reporting clinical results of the PRK performed with the 1 kHz frequency solid-state laser, we obtained good results as to efficacy and safety [4,8].

Tsiklis et al. and Felipe et al., in both their studies on 10 eyes, showed good results at 1-year follow-up after PRK [9,13]. Allan et al. reported visual improvement after the treatment of irregular astigmatism in 14 eyes [10]. Ng-Darjuan et al. used the laser to correct residual refractive errors after previous LASIK procedures with good clinical outcomes, whereas Quito et al. reported good efficacy and safety in 34 eyes treated for hyperopia [11,12].

Shah et al. reported results of the LASEK technique using mitomycin C (MMC) 0.02% for 30 s in myopic eyes with predicted ablation greater than 75 microns with the Pulzar Z1 solid-state laser (CustomVis) [18]. The efficacy index calculated in myopic eyes was similar to our results (1.01 vs. 1.16), with 89% of eyes within 0.5 D, which was 91% in our sample.

Piñero et al. performed the LASIK procedure with the Pulzar Z1 laser on 60 eyes of 34 patients, and reported 96% of eyes being within 0.5 D after the follow-up between 6 and 13 months [19].

In other studies, Piñero et al. showed very good results in the correction of astigmatism and hyperopia with the LASIK technique using the Pulzar Z1 laser [20,21].

Kymionis et al. reported a case of transepithelial phototherapeutic keratectomy with a 213 nm solid-state laser with corneal collagen cross-linking for a patient with keratoconus; follow-up after 6 months presented no complications and haze [22].

In a very recent study, Pajic et al. presented 6-month results of femtosecond lasik performed in five eyes of three patients with a new solid-state laser AquariuZ (Ziemer Ophthalmic Systems AG, Port, Switzerland), reporting encouraging outcomes and confirming growing interest in solid-state technology [24].

In this study, we report the first clinical results of PRK performed with the 210 nm, 2 kHz frequency solid-state laser LaserSoft (Katana Technologies GmbH, Kleinmachnow, Germany).

We evaluated the efficacy, safety, predictability, and stability of the refractive procedure performed in 34 eyes for myopia and compound myopic astigmatism with a 12-month observational period.

As a result of the data analysis, the treatment proved effective and safe in our examined sample, with a gain of lines of visual acuity in 64% of eyes (Figure 1C). No loss of visual acuity lines was registered when preop versus postop CDVA was analyzed (Figure 1C). The predictability of the treatment was good, with 33 eyes (97%) within ±1 D and 29 eyes (91%) within ±0.50 D after 12 months. In eyes with a high SE ± −7 D, a slight overcorrection was registered in the first month of examination. Similar data were reported by Tsiklis et al., who registered overcorrection after PRK in the eyes with high myopia and myopic astigmatism treated with a 213 nm solid-state laser [9].

Similarly, in the excimer laser treatments, the PRK exhibits minor efficacy and predictability in the high refractive errors as compared to the results obtained in low ametropias [17]. In our study, one eye (3%) with high myopia showed the overcorrection of 1D after 12 months of follow-up. The mean SE after 12 months from the treatment was −0.01 D, confirming the good clinical outcome of the procedure. Transient haze (grade 1) was present 1 month after the treatment.

In conclusion, PRK with the solid-state laser used in this study proved good efficacy, safety, stability, and predictability after the 12-month follow-up, confirming the validity of solid-state technology as an alternative for excimer lasers. The limitation of this study is undoubtedly related to the retrospective design and the limited number of patients within the sample.

Further studies on a larger number of patients with long-term follow-up will help us to better define the clinical results achievable with the LaserSoft solid-state technology platform.

## Figures and Tables

**Table 1:** Preoperative characteristics of examined patients.

No. of Eyes (R/L)	34 (15/19)
Sex (M/F)	12/7
Age (y)	34.32 ± 8.27 (21–52)
Refractive errors (D)-sphere	−4.56 ± 2.13 (−8.50 to −1.00)
Refractive errors (D)-cylinder	−0.68 ± 0.87 (−4.00 to 0.00)
Refractive errors (D)-spherical equivalent	−4.90 ± 2.11(−8.63 to −1.25)
logMAR CDVA	−0.03 ± 0.06 (−0.20 to 0.10)
logMAR UDVA	1.20 ± 0.43 (0.05 to 1.70)
Central corneal thickness (CCT)	545.87 ± 33.4 (475–599)
